# Environmental Risks to Public Health in the United Arab Emirates: A Quantitative Assessment and Strategic Plan

**DOI:** 10.1289/ehp.1104064

**Published:** 2012-02-22

**Authors:** Jacqueline MacDonald Gibson, Zeinab S. Farah

**Affiliations:** 1 Department of Environmental Sciences and Engineering, Gillings School of Global Public Health, University of North Carolina–Chapel Hill, Chapel Hill, North Carolina, USA; 2 London School of Economics and Political Science, University of London, London, United Kingdom

**Keywords:** environmental burden of disease, environmental priorities, risk assessment, strategic planning

## Abstract

Background: Environmental risks to health in the United Arab Emirates (UAE) have shifted rapidly from infectious to noninfectious diseases as the nation has developed at an unprecedented rate. In response to public concerns over newly emerging environmental risks, the Environment Agency–Abu Dhabi commissioned a multidisciplinary environmental health strategic planning project.

Objectives: In order to develop the environmental health strategic plan, we sought to quantify the illnesses and premature deaths in the UAE attributable to 14 environmental pollutant categories, prioritize these 14 risk factors, and identify interventions.

Methods: We estimated the disease burden imposed by each risk factor using an attributable fraction approach, and we prioritized the risks using an empirically tested stakeholder engagement process. We then engaged government personnel, scientists, and other stakeholders to identify interventions.

Results: The UAE’s environmental disease burden is low by global standards. Ambient air pollution is the leading contributor to premature mortality [~ 650 annual deaths; 95% confidence interval (CI): 140, 1,400]. Risk factors leading to > 10,000 annual health care facility visits included occupational exposures, indoor air pollution, drinking water contamination, seafood contamination, and ambient air pollution. Among the 14 risks considered, on average, outdoor air pollution was ranked by the stakeholders as the highest priority (mean rank, 1.4; interquartile range, 1–2) and indoor air pollution as the second-highest priority (mean rank 3.3; interquartile range, 2–4). The resulting strategic plan identified 216 potential interventions for reducing environmental risks to health.

Conclusions: The strategic planning exercise described here provides a framework for systematically deciding how to invest public funds to maximize expected returns in environmental health, where returns are measured in terms of reductions in a population’s environmental burden of disease.

The United Arab Emirates (UAE) has modernized more rapidly than any nation in world history. In the 40 years since its founding, the UAE has grown from a primarily nomadic and subsistence fishing population of < 400,000 to a multicultural population of > 4.4 million with a diverse industrial base (Cassen 1978; United Nations 2007). It is home to two international urban centers, Dubai and Abu Dhabi, the latter of which is now, by some measures, the world’s wealthiest city (Gimbel 2007). This rapid development has been made possible by oil exports: The UAE owns approximately 8% of the world’s remaining oil reserves, most contained in the emirate of Abu Dhabi, the largest of the UAE’s seven emirates (U.S. Energy Information Administration 2010). With 97.8 billion barrels of proven reserves (five times the remaining U.S. supply), the UAE ranks sixth among all countries in future oil production potential (U.S. Energy Information Administration 2009, 2010).

The UAE’s rapid modernization has brought remarkable advancements in public health. For example, during 1970–1975, female life expectancy averaged 46.5 years (Cassen 1978); by 2005–2010 it had increased by > 75%, to 81.5 years—higher than that in the United States (United Nations 2007). However, modernization also has brought new risks. As infectious disease rates have declined, disease patterns have shifted to resemble those in developed countries, with increases in noninfectious conditions such as cardiovascular diseases, cancer, and diabetes. Some of this disease burden may be attributable to environmental pollution that has accompanied the nation’s economic growth. Fear about environmental risk factors (whether or not such fear is warranted on technical grounds) is increasing, as evidenced by frequent reporting on environmental risks in the local news media, including headline news stories that have raised alarms. To address these concerns, in 2007 the Environment Agency–Abu Dhabi (EAD) issued a request for proposals (RFP) for a study to quantify the environmental burden of disease in the UAE and develop a strategic plan for reducing preventable, environmentally triggered diseases. The RFP also required an epidemiological study, the specific nature of which was to be proposed by respondents to the RFP. A consortium led by the University of North Carolina–Chapel Hill Gillings School of Global Public Health and including UAE University, RAND, Resources for the Future, and the Norwegian Institute for Air Quality Research was awarded the contract to carry out this project. The project is the first of its kind in the Middle East region and is perhaps the most ambitious national-level effort to quantify the environmental burden of disease, prioritize environmental risks to health, and develop a strategy for reducing those risks that any nation has yet undertaken. The World Health Organization (WHO) Centre for Environmental Health Activities in Amman, Jordan, which provided project oversight, envisages the methods used and results from this work as models for future, similar endeavors in the Middle East and other regions.

Although some public health specialists have defined environmental risks to health in broad terms (Smith et al. 1999), at the request of the EAD and the WHO we focused on risks of exposure to harmful chemical, physical, and biological agents released to the environment through human activities. Specifically, we considered 14 risk factors: *a*) ambient air pollution, *b*) indoor air pollution (including environmental tobacco smoke), *c*) drinking water contamination, *d*) surface water pollution (in this case, only coastal water because the UAE has no other surface water resources), *e*) soil and groundwater pollution from solid and hazardous waste disposal, *f*) seafood contamination, *g*) contamination of fruits and vegetables with pesticides, *h*) ambient noise, *i*) stratospheric ozone depletion and resulting risks of excess exposure to ultraviolet radiation, *i*) electromagnetic fields from power lines, *j*) occupational exposures in the industrial sector, *k*) occupational exposures at construction sites, *l*) occupational exposures in agriculture, and *m*) global climate change.

Here we summarize our approach for developing the UAE environmental health strategic plan and the results of the planning process. The method combines quantitative risk assessments with a structured stakeholder deliberation process tested in previous empirical research.

## Methods

The method we used builds on the strengths and seeks to avoid the pitfalls of previous environment and health plans from other countries. We systematically reviewed publicly available national environmental health planning documentation from Europe, Australia, and China [as summarized in Supplemental Material, Table 1 (http://dx.doi.org/10.1289/ehp.1104064)]. In addition, although the United States has never produced a formal environmental health strategy document, the U.S. Environmental Protection Agency (EPA) has previously sought to set priorities on environmental risks to health (U.S. EPA 1987, 1990), and we reviewed documentation from these exercises. We found that most countries used ad hoc priority-setting and planning methods, with little to no underlying systematic risk analysis. We also found that many plans were developed entirely by government agency personnel, without engaging the wider stakeholder community whose cooperation would be needed to implement the resulting plan. The topics addressed in and content of the plans also varied widely, ranging from general statements about the importance of considering environmental health impacts in decisions in multiple sectors to detailed documents describing specific new environmental regulations and implementation time lines.

**Table 1:** Estimated disease burden due to selected environmental risk factors in the UAE.

Exposure route	Risks evaluated for this project	Exposure indicators	Adverse health conditions	Attributable fatalities in 2008 (95% CI)	Attributable health care facility visits in 2008 (95% CI)
Air (breathing)		Ambient (outdoor) air pollution		PM10, daily average (μg/m3) PM2.5, annual average (μg/m3) Ground-level ozone, daily (24-hr) average (ppb) Ground-level ozone, annual average of daily maximum concentration (ppb)		All-cause mortality (all ages) and respiratory mortality (< 5 years) Respiratory and cardiovascular morbidity (all ages) All-cause, cardiopulmonary, and lung cancer mortality (> 30 years) Total nonaccidental, cardiovascular, and respiratory mortality (all ages) Respiratory morbidity (all ages) Respiratory mortality (> 30 years)		650 (140, 1,400)		15,000 (5,000, 27,000)
						
						
						
						
						
						
		Indoor air pollution in residential environments		PM10, PM2.5 Benzene, formaldehyde Radon Environmental tobacco smoke Bioaerosols (mold) Incense use		Asthma (< 5 years) Asthma (< 3 years) Lung cancer Lung cancer and lung cancer mortality, leukemia, cardiovascular disease and cardiovascular disease mortality, asthma (< 18 years), lower respiratory tract infection (< 6 years) Childhood (6–12 years) and adult asthma Respiratory tract cancer and respiratory tract cancer mortality		290 (110, 540)		89,000 (42,000, 140,000)
						
						
						
						
						
Water (drinking, bathing, inhaling droplets)		Drinking water contamination		Disinfection by-products Microbial contamination		Bladder, rectal, and colon cancer Gastroenteritis		12 (8, 16)		46,000 (15,000, 61,000)
						
		Coastal water pollution		Microbial contamination		Gastroenteritis		0		2,300 (1,400, 3,300)
Soil (dermal contact followed by ingestion)		Soil and associated groundwater pollution due to solid and hazardous waste disposal		Heavy metals, hydrocarbons, pesticides		Cancers, neurological disorders, adverse pregnancy outcomes		NA		NA
Food (eating)		Seafood contamination		Methylmercury		Neurological disorders		4 (0, 10)		27,000 (0, 67,000)
		Pesticides on fruits and vegetables		Pesticides		Pesticide poisoning		0 (NA)		0 (0, 89,000)
Sound and electromagnetic radiation (contacting in ambient environment)		Ambient noise above healthful levels		Noise > 65 dBA		Stress, sleep loss, decreased cognitive performance		0 (NA)		NA
		UV radiation above natural levels as a result of stratospheric ozone depletion		UV radiation		Cancers of skin and eyes, corneal damage, cataracts		20 (16, 24)		15,000 (12,000, 18,000)
		Electromagnetic fields from power lines						0 (0, 4)		2 (0, 14)
Occupational environments		Industry		Carcinogens and leukemogens PM Noise		Lung cancer, leukemia, malignant mesothelioma Asthma, chronic obstructive pulmonary disease, asbestosis, silicosis Hearing loss		10 (5, 20)		79,000 (NA)
						
						
		Construction		Same as for industry		Same as for industry		15 (10, 30)		120,000 (NA)
		Agriculture		Same as for industry		Same as for industry		65 (0, 100)		60,000 (0, 360,000)
Global climate change		Global climate change		Heat exposure		Cardiovascular disease		3 (0, 6)		410 (84, 800)
Abbreviations: NA, not applicable; PM2.5, PM ≤ 2.5 μm in aerodynamic diameter; PM10, PM ≤ 10 μm in aerodynamic diameter; UV, ultraviolet. These estimates were updated after the risk ranking exercise. They also have been updated, with newer health outcome data, since publication of Li et al. (2010). Full details on these estimates, as well as the preliminary estimates presented during the risk ranking exercise, are reported elsewhere (MacDonald Gibson et al., in press).										

From our review of these plans, it became clear that no best international practice for environmental health strategic planning has yet emerged. A consensus report developed by participants in an international workshop on national environmental health planning reached a similar conclusion and recommended a systematic review of differences in the methods used (Briggs et al. 1999). To our awareness, such a review has not yet been completed.

Based on our review, we developed a five-step planning process that quantifies the burden of disease attributable to each risk factor and systematically engages stakeholders in prioritizing the risk factors and identifying potential interventions. The five steps are described below.

*Step 1: quantify risks.* We quantified the public health risks associated with the 14 risk factors we were asked to consider. Our estimates of attributable deaths and illnesses follow the methods for environmental burden of disease quantification developed by the WHO, which in turn are derived from long-standing principles of public health (WHO 2003a, 2003b, 2003c, 2004a, 2004b, 2004c, 2004d, 2004e, 2004f, 2004g, 2005a, 2005b, 2006, 2007a, 2007b, 2008). In brief, the fraction of observed deaths or illnesses in a population attributable to an environmental exposure is estimated as


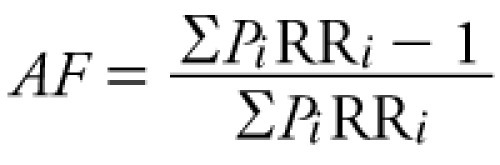
[1]

where *AF* is the attributable fraction; *P_i_* is the proportion of the population at exposure category *i*, including the unexposed population; and RR*_i_* is the relative risk at exposure category *i*, compared with the reference level (Σ*P_i_*RR*_i_ = P_1_*RR*_1_ + P_2_*RR*_2_ + . . . + P_unexposed_ ×* 1).

The total numbers of deaths and illnesses attributable to each risk factor are then obtained by multiplying *AF* by the observed deaths or illnesses for each relevant health end point in the applicable population:

*D*_attrib_ = *AF*(*D*_total_), [2]

where *D*_attrib_ is the attributable disease burden and *D*_total_ is the total disease burden in the population.

Our estimates of *D*_total_ are based on complete death records and medical insurance records from 2008 provided by the Health Authority–Abu Dhabi. Relative risk estimates were drawn from a comprehensive search of the epidemiological literature. Estimates of population exposures to specific pollutants were obtained from a combination of local data, modeled estimates, and pertinent literature. Full details on the methods and results are documented elsewhere (MacDonald Gibson et al., in press); details on risk estimates for ambient air pollution, seafood contamination, and pesticide residues on fruits and vegetables were described previously by Li et al. (2010) and Davidson et al. (2011).

We encoded the risk calculations using Analytica software (version 4.1; Lumina Decision Systems Inc., Los Gatos, CA, USA).The resulting simulation model, which we named the UAE Environmental Burden of Disease Model, will facilitate future efforts to estimate the effects of interventions that reduce environmental pollutant concentrations or exposures on the environmental burden of disease. Uncertainty and variability in model input variables were represented using probability distributions, and uncertainty in the model output was estimated through statistical simulation. The complete model, including all input parameters and probability distributions used to represent them, is described elsewhere (MacDonald Gibson et al., in press).

*Step 2: prioritize risks.* To prioritize the 14 risks, we used the deliberative method for ranking risks, which emerged from research commissioned by the U.S. Office of Science and Technology Policy to address criticisms of U.S. EPA environmental risk ranking exercises conducted in the 1980s (Davies 1996; DeKay et al. 2001; Florig et al. 2001; Long and Fischhoff 2000; Morgan KM et al. 1999; Morgan MG et al. 2000; Willis et al. 2004, 2010). The method incorporates both quantitative risk information and stakeholder deliberations in a systematic process that proceeds as follows. First, risk analysts prepare risk summary sheets for the risks of concern. These summaries characterize the risks according to 12 metrics that experts in risk perception have determined through experimental research (e.g., Slovic et al. 1980) to reflect the key concepts individuals consider when they rank risks: *a*) number of deaths per year; *b*) chance in a million of death per year for the average resident; *c*) chance in a million of death per year for the resident at highest risk; *d*) greatest number of deaths in a single event; *e*) serious long-term illnesses each year; *f*) less serious long-term illnesses each year; *g*) serious short-term illnesses per year; *h*) less serious short-term illnesses per year; *i*) time between exposure and health effects; *j*) quality of scientific understanding; *k*) uncertainty in risk estimates; and *l*) ability of an individual to control exposure to the risk.

Next, project managers recruit a diversity of stakeholders to participate in day-long focus group sessions of 8–12 participants each. Participants rank risks based on information in the summary sheets, both individually and as a group. Finally, analysts compile statistical information about the rankings. Ideally, many such focus groups are convened, allowing statistical comparisons of differences between groups as well as between individuals.

This method was extensively tested and refined in a set of experiments involving 218 professional risk managers (Florig et al. 2001). Since then, a variety of national and international entities have used the method in risk-ranking exercises. For example, in Canada, the Consumer and Market Demand Agricultural Policy Research Network used the method to rank food safety risks (Webster et al. 2008). The U.S. Army Corps of Engineers is using the method to rank hurricane mitigation opportunities on the Louisiana Gulf Coast (U.S. Army Corps of Engineers 2007). To our knowledge, our project is the first to employ the method as part of a national environmental strategic planning exercise—even though the method was initially developed with that purpose in mind. Full details on method implementation in the UAE have been reported previously (Willis et al. 2010).

*Step 3: identify candidate initiatives for reducing risks and measuring progress.* To be consistent with the *Abu Dhabi Government Strategic Planning Handbook* (Abu Dhabi Executive Council 2007), the strategic plan needed to express recommendations in terms of initiatives, that is, specific steps that can be taken in the next 4–20 years to reduce risks and/or improve understanding of options for controlling the risks. It also needed to include key performance indicators for measuring progress.

Scientists on our team who had expertise relevant to each risk area developed initial lists of candidate initiatives and key performance indicators. We compiled their suggestions in worksheets listing the risk factor, potential initiatives for decreasing risks, and key performance indicators for measuring progress, along with descriptions of these indicators, their units of measurement, and suggested methods for assessing the levels of each indicator over time. The worksheets also included space for stakeholder comments.

*Step 4: solicit stakeholder feedback and new ideas.* We held two 1-day workshops with stakeholders to review, discuss, and revise the lists of initiatives and key performance indicators. Of the 63 participants, 46 represented emirate-level and local government agencies, including the emirate-level environmental, public health, food quality, and transportation agencies. Also present were 10 scientists from universities in the UAE, 2 industry representatives, 2 consultants, and 3 representatives of federal agencies. (In the UAE, in many respects, especially in Abu Dhabi and Dubai, emirate-level agencies are preeminent over their federal counterparts.)

Participants divided into focus groups based on priority risk area. Participants self-sorted into the groups based on their expertise. Facilitators for each group began the first workshop by reviewing the nature of the risk, the goals for the workshop, and the suggested initiatives and key performance indicators. After discussion, stakeholders then deleted from the worksheets ideas that already were under way in the UAE or that were infeasible for political or cultural reasons and added ideas beyond those provided by our team’s scientists. In the months between the first and second sets of workshops, participants were sent revised worksheets and asked to provide comments. During the second round of workshops, participants reviewed the revised initiatives and performance measures and made additional changes. Participants then were allowed 3 weeks after the second workshop to provide final comments.

*Step 5: prepare strategy and action plan document.* We documented the outcome of the risk ranking and strategic planning exercises in a formal document, the *National Strategy and Action Plan for Environmental Health, United Arab Emirates—2010* (Environment Agency-Abu Dhabi et al. 2009). The document summarizes basic information about each priority risk and then lists the recommended initiatives and key performance indicators. Also included for each initiative is a listing of stakeholder groups that should be involved in implementation.

## Results and Discussion

*Quantification of environmental burden of disease.* All told, there were 8,865 reported deaths and 1.7 million health care facility visits (from any cause) in the UAE in 2008. The 14 environmental risk factors that we considered may contribute to hundreds to thousands of annual premature deaths and tens to hundreds of thousands of annual illnesses. According to our estimates, the leading contributors to premature death are ambient and indoor air pollution, to which 650 [95% confidence interval (CI): 140, 1,400] and 290 (95% CI: 110, 540) annual deaths may be attributed, respectively (Table 1). The leading contributor to excess medical visits is exposures in the construction industry.

Taken out of context, our risk estimates may cause alarm. In fact, earlier versions of our estimates appeared on the front page of local newspapers, prompting highly placed individuals to contact EAD for an explanation. However, it is important to realize that the environmental burden of disease in the UAE is comparable to that in Western industrialized nations and is quite low by global standards. As an example, according to our estimates, the annual incidence of deaths attributable to ambient air pollution in the UAE is 0.14 per 1,000 people. In contrast, the WHO (2009) estimates the incidence of death due to particulate matter (PM) in ambient air per 1,000 people to be 0.19 in the United Kingdom, 0.19 in Japan, and 0.14 in the United States. Thus, the UAE’s burden of mortality due to ambient air pollution is less than that in the United Kingdom and Japan and is similar to that in the United States.

**Figure 1:**
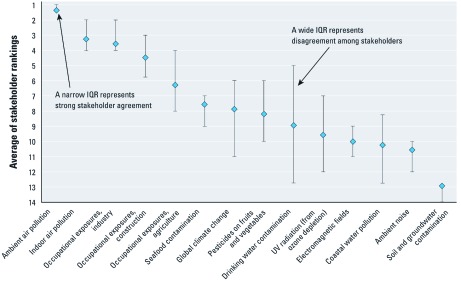
Summary of final individual risk rankings. A rank of 1 indicates the highest priority. Diamonds represent the average of the 56 individual rankings, and the bars show the interquartile range (IQR; 75th and 25th percentiles) of the rankings. The width of these percentile bars is a measure of the level of agreement or disagreement about the priority of the particular risk. Adapted from Willis et al. (2010).

*Ranking of environmental risks to health.* The risk-ranking exercise revealed strong agreement among participants on both the highest- and lowest-priority risks, as described in detail by Willis et al. (2010). Information about the individual rankings collected from the 56 stakeholders who submitted final individual rankings are summarized in Figure 1. (In all, 73 stakeholders participated, but only 56 stayed for the full day to submit final rankings.) Stakeholders agreed almost unanimously that the highest priority risk from among the 14 risk categories considered is outdoor air pollution (mean rank, 1.3 out of 14). On average, stakeholders ranked indoor air pollution as second most important (mean rank, 3.3). Occupational exposures in industry ranked third (mean rank, 3.6). Also worth noting is that stakeholders disagreed on the relative importance of several of the risks, most notably drinking water contamination. Although the mean rank for drinking water contamination was 9.2, 5 participants ranked it as second most important, whereas 4 ranked it as least important of the 14 risks considered. Those who ranked drinking water as a low priority stressed that the UAE’s water supply is highly treated with advanced desalination systems that remove all contaminants and, further, that many residents drink bottled water. Those who rated water as a high priority emphasized the importance of water resources in the arid UAE, which receives < 4 inches of rainfall a year, and they pointed out that many residents must store their household water in rooftop storage tanks because of intermittent delivery of piped water, leaving the desalinated water vulnerable to recontamination. Stakeholders also disagreed about the relative importance of climate change, pesticide contamination of produce, stratospheric ozone depletion, and coastal water pollution (where the focus was on exposure to contaminants during swimming and other water-based recreational activities).

Consistent with previous pilot studies of the deliberative risk ranking method, results were very similar from one focus group to the next. In all, we held five 1-day focus groups, each involving 8–20 people. All of the groups ranked ambient air pollution as the highest risk and residential soil pollution as the lowest or second lowest risk (Willis et al. 2010). In all groups, indoor air quality and occupational exposures in industry and construction were ranked in the top six. Also consistent with previous pilot studies, agreement among individuals increased after the group discussions, with mean pairwise correlations among individual rankings increasing by statistically significant amounts in four of the five groups (Willis et al. 2010).

Surveys administered after the focus groups showed a high level of satisfaction with the process. Most participants were satisfied or very satisfied with the group ranking, and most approved or strongly approved of submitting the group rankings to the EAD for use in making decisions. On a scale of 0–6, with 0 representing “strongly disapprove” and 6 representing “strongly approve,” the average response to the question “How strongly would you approve of submitting your group’s rankings to EAD for use in making decisions?” was 4.36, with 70% providing a response ≥ 4 (Willis et al. 2010).

Based on the risk ranking results and follow-up discussions with the EAD and WHO, the following eight risk areas were retained for subsequent analysis: *a*) outdoor air pollution; *b*) indoor air pollution; *c*) occupational exposures in industry, construction, and agriculture; *d*) global climate change; *e*) drinking water contamination; *f*) coastal water pollution; *g*) soil and groundwater contamination due to solid and hazardous waste; and *h*) contamination of produce and seafood with environmental pollutants. We retained for analysis some risk factors that ranked low, on average, because of disagreement among stakeholders about the importance of the problem. For example, although coastal water pollution ranked 12th on average, one group ranked it as among the top seven risk factors. We retained some other risk factors because the EAD and WHO expressed concern that although the factor might not be a current priority, it could become so in the future if the UAE continues to develop at its current, rapid pace. For example, we retained soil pollution on the list (even though it ranked lowest) because of plans for extensive future residential development in areas currently containing large numbers of uncontrolled waste disposal sites (primarily in the western region of Abu Dhabi).

*Strategy and action plan.* The two strategic-planning workshops produced consensus lists of initiatives for the UAE to pursue to reduce health risks for each of the eight remaining risk areas. In total, the *National Strategy and Action Plan for Environmental Health, United Arab Emirates—2010* (MacDonald Gibson and Brammer 2009) identifies 216 potential initiatives. Within each risk area, the initiatives are organized according to six cross-cutting goals (called “targets,” to be consistent with the Abu Dhabi strategic planning guidance): *a*) reduce pollutant levels and human exposure to pollutants, *b*) improve data quality and availability, *c*) improve scientific understanding of environmental health risks, *d*) build sustainable human and institutional capacity, *e*) support urban development that promotes environmental health, and *f*) improve environmental awareness.

The strategic plan includes key performance indicators (a total of 179) for measuring progress in each risk area. For example, key performance indicators for indoor air quality include the percentage of public buildings in various categories that enforce indoor smoking bans and the percentage of buildings of various types exceeding radon thresholds. An example key performance indicator for drinking water is the percentage of water samples collected from points of use that comply with potable water quality standards.

A sample page from the 100-page strategic plan is provided in Supplemental Material, Figure 1 (http://dx.doi.org/10.1289/ehp.1104064). Each recommended initiative is given a tracking number (e.g., EH-3/T-1/I-2) to enable the EAD to track progress in a manner consistent with Abu Dhabi strategic planning guidance documents. Along with each recommendation are a list of government agencies that should be involved in implementation and a suggested time line.

The EAD had only 1 year to produce the risk ranking and strategic plan—too short a time period to prioritize and assess the cost-effectiveness of the 216 recommended initiatives in detail. The UAE Environmental Burden of Disease Model (described in “Step 1: quantify risks”) is intended to help future UAE policy analysts compare public health benefits among the recommended initiatives for finer scale prioritization. The model can be used to estimate how a change in pollutant levels or a change in the size of the exposed population would affect the burden of disease within each risk area.

Ideally, the UAE will view the strategic plan as a dynamic document, to be revised on a regular basis as cost–benefit analyses reveal the most promising initiatives, new data reduce uncertainties in the most important contributors to the environmental disease burden, and conditions continue to change in the country.

## Conclusions

Empirical research on methods that lead to the most effective strategic plans in the public sector—ones that actually cause positive change—is lacking (Steurer and Martinuzzi 2005). Even in the private sector, where success can be measured in terms of profitability, there is a shortage of information on the best planning approaches, and the effectiveness of strategic planning as a means of increasing profits is a matter of debate (e.g., Bowman and Helfat 2001; Falshaw et al. 2006; Miller and Cardinal 1994). Nonetheless, we believe the priority-setting process we used in the UAE provides a sound model for future state- and national-level environmental strategic planning. Just as financial managers seek to balance risks and benefits through systematic analysis, this planning exercise provides a framework for systematically deciding how to invest funds to maximize expected returns in environmental health—where returns are measured in terms of decreases in overall population disease burden. Indeed, Honoré et al. (2010) argued that the use of formal decision analysis techniques such as we used in the UAE should be applied more widely in the allocation of resources for public health. They noted that public health agencies “develop budgets to allocate funding perceived as based on need, but traditionally, lacking quantitative analysis to support these complicated decisions” (Honoré et al. 2010).

In an ideal scenario, after the deliberative method for ranking risk factors reveals which risk areas are most important to stakeholders and after menus of possible risk reduction initiatives are developed, risk analysts would estimate the effects of each initiative on each of the risk attributes in the risk summary sheets. Analysts also would estimate the total costs of each initiative and develop measures of cost effectiveness (e.g., cost per life saved and cost per avoided case of specific illnesses). Then, focus groups, each addressing one risk area, could prioritize the initiatives, based on their effects on the risk attributes as well as cost-effectiveness, using a process analogous to the deliberative method for ranking risks. Results across individuals and focus groups could be compared to identify areas of clear agreement or strong disagreement. Strong agreement could be considered as a mandate for moving forward rapidly on the initiative.

Beyond identifying priority initiatives, the key to the ultimate success of this and any other strategic planning effort is implementation. Even the best-conceived plan will fail to yield its anticipated benefits unless some authority—whether an agency or interagency group—is empowered with implementation authority (Steurer and Martinuzzi 2005). Toward this end, in October 2011 the EAD and the Health Authority–Abu Dhabi began meeting regularly to follow up on the recommendations of the strategic plan; they intend eventually to engage other key agencies and stakeholders in these meetings.

Overall, the UAE strategic plan was received with enthusiasm among the many stakeholders who participated in its development and the highest levels of government in the UAE. We believe the process we used to prepare this plan can serve as a useful model for other nations undertaking systematic environmental health strategic planning exercises—to work toward the goal of maximizing the overall social benefits of government and private-sector investments in protecting public health from undue environmental risks. Building on the deliberative method for ranking risks, our approach overcomes one of the most important limitations (lack of a systematic process for combining quantitative risk analysis with stakeholder engagement) of previous environmental health strategic planning exercises around the world.

## Supplemental Material

(442 KB) PDF
