# Supplementary material for: Environmental Risks to Public Health in the United Arab Emirates: A Quantitative Assessment and Strategic Plan
Source: Environ Health Perspect. 2012 Feb 22;120(5):681–6. doi: 10.1289/ehp.1104064 (PMC3346776; doi:10.1289/ehp.1104064)
Supplement: (442 KB) PDF [file ehp.1104064.s001.pdf]

Supplemental Material

Environmental Risks to Public Health in the United Arab Emirates:

A Quantitative Assessment and Strategic Plan

Jacqueline MacDonald Gibson[1*] and Zeinab S. Farah[2]
[1]Department of Environmental Sciences and Engineering
Gillings School of Global Public Health
University of North Carolina
Chapel Hill, NC, USA

[2]London School of Economics and Political Science
University of London
London, United Kingdom

Supplemental Material, Table 1. National Environmental Health Strategy and Action Plan Documents

Country	Document Title (Year)	Strategic Planning Approach	Quantitative Risk Information Used?	Stakeholders Involved?	References
Albania	National Environmental Health Action Plan (1998)	Working group of experts from government agencies and research institutes prepared qualitative assessments of environmental risks to health and developed recommended actions to address these risks.	No	No	Ministry of Health 1998
Austria	Austrian National Environmental Health Action Plan (1999)	Representatives from three federal ministries assessed available information on environmental risks to health and developed possible solutions.	No	No	Federal Ministry of Environment, Youth, and Family Affairs et al. 1999
Australia	National Environmental Health Strategy (1999)	Representatives of national, state, and territorial public health agencies collaborated to write the strategy.	No	Limited—stakeholders invited to review strategic plan document	Commonwealth of Australia 1999
China	China National Environment and Health Action Plan 2007-2015 (2007)	Method for developing the plan is not clear from the available documentation. Listed as “subscribing departments” are 18 government agencies, from the Ministry of Health, to the State Environmental Protection Administration and State Administration of Radio Film and Television.	No	Not clear from documentation	Ministry of Health of China 2007
Czech Republic	National Environmental Health Action Plan (1998)	Working group of experts representing seven government organizations assessed existing information on environmental quality and health outcomes and based priorities on their judgments of this information.	No	No	Cizkova et al. 1999
Estonia	The National Environmental Health Action Plan of Estonia (1999)	A group of 37 experts from government agencies and research institutes conducted quantitative risk assessments. Recommended actions were prioritized based on whether risks in Estonia are higher than in other European nations.	Yes	Limited—stakeholders represented government agencies and research centers	Health Protection Department, Ministry of Social Affairs 1999
Finland	Finnish Environmental Health Action Plan (1999)	Stakeholder committee quantified deaths and illnesses attributable to specific environmental risks. Met 18 times to prioritize risks and develop objectives and actions.	Yes	Yes	Finnish Environmental Health Committee 1997

Supplemental Material, Table 1 (cont.)

Country	Document Title (Year)	Strategic Planning Approach	Quantitative Risk Information Used?	Stakeholders Involved?	References
France	National Environment and Health Action Plan 2004-2008 (2004)	Expert group prepared report that analyzed exposure to environmental risks and made recommendations for action priorities.	Yes	Limited—stakeholders invited to comment on proposed actions	Ministere de la Sante et de la Protection Sociale et al. 2004
Lithuania	National Environmental Health Action Plan Lithuania (2001)	Thirteen working groups of experts developed recommended actions based on qualitative assessment of environmental health risks.	No	Limited—involvement limited to experts	Ministry of Health and Ministry of Environment 2001
Malta	National Environmental Health Action Plan (2006)	The Ministry of Health, the Elderly, and Community Care developed recommended actions based on a qualitative assessment of environmental conditions.	No	No	Environmental Health Policy Co-Ordination 2006
Netherlands, The	Environmental Health Action Plan: Implementing More Powerful Policy (2002)	Stakeholder working groups assessed bottlenecks and gaps in existing environmental health policies and broad actions to address the identified needs.	No	Yes	Ministry of Housing, Spatial Planning and the Environment and Ministry of Health, Welfare and Sport 2002
Poland	Long-Term Programme: Environment and Health (2001)	Broad outline of steps needed to quantify the environmental burden of disease, formulate environmental policies, and establish medical prevention programs was developed by the Ministry of Health and Ministry of Environment.	No	No	Ministry of Health and Ministry of Environment Poland 1999; Cizkova et al. 1999
Romania	The Romanian National Environmental Health Action Plan (1997)	Prepared by a steering committee composed of representatives from seven government agencies, academic institutions, and nongovernment organizations. Proposed actions were solicited from a wide variety of stakeholders through use of formal “action proposal forms.”	No	Yes	Cizkova et al. 1999
Slovak Republic	National Environmental Health Action Plan for the Slovak Republic II (2000)	Experts in public health developed the plan based on an analysis of the implementation of a 1997 action plan. The group considered trends in environmentally related health effects and pollutant levels in the environment but did not quantify risks.	No	No	Government of the Slovak Republic 2000

Supplemental Material, Table 1 (cont.)

Country	Document Title (Year)	Strategic Planning Approach	Quantitative Risk Information Used?	Stakeholders Involved?	References
Sweden	Environment for Sustainable Health: An Action Plan for Sweden (1996)	Work group of 30 scientists and government officials quantified risks and then ranked them as high, medium, or low priority based on estimated number of health outcomes per year due to the risk.	Yes	Limited—no industry groups or non-government organizations involved	Victorin et al. 1999
Switzerland	Sustainable Development: Action Plan Environment and Health (1997)	Interagency working group identified and ranked 17 areas where additional measures to protect environmental health are needed. Group then identified potential interventions and goals (targets).	No	Yes	Kahlmeier et al. 2002; Swiss Federal Office of Public Health and Swiss Agency for the Environment Forests and Landscape 1997
Ukraine	National Environmental Health Action Plan of Ukraine (1999)	Expert team identified broad categories of environmental risks to health and measures to reduce each risk.	No	No	Ministry of Health Care of Ukraine and Ministry of Environmental Protection and Nuclear Safety of Ukraine 1999
United Kingdom	National Environmental Health Action Plan (1996)	The Department of Environment, Food and Rural Affairs Actions recommended actions based on a qualitative assessment of environmental conditions. This action plan was overtaken by the development of the 1999 U.K. Sustainable Development Strategy.	No	Limited—stakeholders invited to submit comments	Department of Health and Department of Environment 1996; Capleton et al. 1996
Uzbekistan	National Environmental Health Action Plan of the Republic of Uzbekistan (1999).	A stakeholder working group recommended actions based on a qualitative assessment of the current state of environmental health and of measures already in place to reduce environmental health risks.	No	Yes	State Committee of Environment of the Republic of Uzbekistan and Ministry of Health of the Republic of Uzbekistan 1999

Occupational Exposures (EH-3):

Initiatives to Reduce the Burden of Disease Due to Occupational Exposures

Target 1: Reduce pollutant levels and human exposure to pollutants	Stakeholders
**EH-3 / T-1 / I-1**<br>Establish a clearly defined higher federal committee and local authorities (e.g., Occupational Health Authority of Abu Dhabi or Dubai) to regulate occupational health and safety (OHS) laws in the UAE.<br>**2010** · 2011 · 2012 · 2013 · 2014	Emirate environment and health agencies<br>MOEW · MOH · MOI · MOL
**EH-3 / T-1 / I-2**<br>Adopt nationwide the Environment, Health and Safety Management System (EHSMS) class 1 indicators adopted by Abu Dhabi (Code of Practice 15—Hazardous Materials, version 1.2, July 2009). In addition:<br>• Identify and assess occupational health workplace hazards and develop a risk management system in line with international standards and best practices.<br>• Implement risk management strategies and controls, applying hierarchy of control principles.<br>• Conduct periodic review and monitoring of the effectiveness of applied occupational-health control measures.<br>**2010** · **2011** · 2012 · 2013 · 2014	Emirate environment and health agencies, along with industry and labor representatives<br>MOEW · MOH · MOL
**EH-3 / T-1 / I-3**<br>Compare the UAE federal labor law with international standards, following the EHSMS regulatory framework. Revise the law as appropriate. In addition:<br>• Establish new standards for exposures relevant to the UAE, where these do not exist presently under UAE law.<br>• Consider developing one federal standard, as a minimum, which must be implemented in all emirates.<br>• Consider the adoption of international occupational exposure standards and biological exposure indices, such as the American Conference of Governmental Industrial Hygienists (ACGIH) threshold limit values, U.K. workplace exposure limits, and German Research Foundation (DFG) standards.<br>**2010** · 2011 · 2012 · 2013 · 2014	Emirate environment and health agencies<br>MOEW · MOH · MOL
**EH-3 / T-1 / I-4**<br>Revise emirate laws regarding OHS and update annually; and harmonize federal and emirate regulations.<br>**2010** · 2011 · 2012 · 2013 · 2014	Emirate environment and health agencies<br>MOEW · MOH · MOI · MOL
**EH-3 / T-1 / I-5**<br>Implement, inspect, and enforce occupational exposure standards.<br>**2010** · **2011** · **2012** · **2013** · 2014	Appropriate emirate agencies<br>MOH · MOL
**EH-3 / T-1 / I-6**<br>Increase the size and competency of work-site inspection teams in relevant federal ministries and emirate authorities.<br>**2010** · **2011** · **2012** · **2013** · 2014	Appropriate emirate agencies<br>MOH · MOI · MOL

Supplemental Material, Figure 1. Example page from the *National Strategy and Action Plan for Environmental Health, United Arab Emirates*. Shown here are some of the recommendations to reduce the risk of occupational exposure to airborne particulate matter, carcinogens, and noise. The "Stakeholders" panel in the shaded box on the right indicates the government agencies recommended for involvement in implementing each recommendation. The numbers (e.g., EH-3/T-1/I-4) are for tracking implementation and are consistent with tracking systems for other Abu Dhabi strategic plans. The green bars below each recommendation show suggested time lines for beginning implementation. Abbreviations: MOEW: Ministry of Environment and Water; MOH: Ministry of Health; MOI: Ministry of the Interior; MOL: Ministry of Labor.

Supplemental Material, References

Capleton, AC, Stevens, J, Harrison, PTC. 2005. The impact of the European environment and health process on UK environment and health policy, plans and practice: what difference has it made? European Journal of Public Health 15(5):546-551.

Cizkova H, Kazmarova A, Dumitrescu A, Janikowski R. 1999. The NEHAP experience in the Czech Republic, Romania and Poland. In: Environmental Health for All: Risk Assessment and Risk Communication for National Environmental Health Action Plans (Briggs DJ, Stern R, Tinker TL, eds). Dordrecht, The Netherlands: Kluwer Academic Publishers, 17-34.

Department of Health and Department of Environment. 1996. The United Kingdom National Environment and Health Action Plan. London: Department of Health.

Commonwealth of Australia. 1999. National Environmental Health Strategy. Publication number 2592. Melbourne: enHealth. http://enhealth.nphp.gov.au/strategy/nehs/index.htm.

Environmental Health Policy Coordination. 2006. National Environmental Health Action Plan Malta. Valletta, Malta: Ministry of Health, the Elderly, and Community Care.

Federal Ministry of Environment Youth and Family Affairs, Federal Ministry of Labour Health and Social Affairs, Bundesministerin fur Frauenangelegenheiten und Verbraucherschutz. 1999. Austrian National Environmental Health Action Plan. Vienna: Federal Ministry of Environment, Youth and Family Affairs.

Finnish Environmental Health Committee. 1997. Finnish Environmental Health Action Plan. Helsinki: Oy Edita Ab.

Government of the Slovak Republic. 2000. National Environmental Health Action Plan for the Slovak Republic II. Resolution: 815/2000. Bratislava: Government of the Slovak Republic.

Haralanova M. 2000. Implementation of National Environmental Health Action Plans in the Czech Republic, Estonia, Lithunia, Poland and the Slovak Republic. European Epi-Marker 4: 2-6.

Health Protection Department. 1999. The National Environmental Health Action Plan of Estonia. Tallinn: Ministry of Social Affairs.

Kahlmeier S, Nunzli N, Braun-Fahrlander C. 2002. The first years of implementation of the Swiss National Environment and Health Cation Plan (NEHAP): lessons for environmental health promotion. Soz.-Praventivmed 47:67-79.

Ministere de la Sante et de la Protection Sociale, Minstere de l'Ecologie et du Developpement Durable, Ministere de l'Emploi, du Travail et de la Cohesion Sociale, Ministere Delegue a la Recherche. 2004. National Environment and Health Action Plan 2004-2008: A New Step Forward in Prevention of Health Effects Due toi Environmental Pollution. Maisons-Alfort, France: ANSES.

Ministry of Health of China. 2007. China National Environment and health Action Plan (2007-2015). Beijing: Ministry of Health of China.

Ministry of Health. 1998. National Environmental Health Action Plan. Tirana, Republic of Albania: Ministry of Health.

Ministry of Health Care of Ukraine and Ministry of Environmental Protection and Nuclear Safety of Ukraine. 1999. National Environmental Health Action Plan of Ukraine 1999-2005. Kiev: Ministry of Health Care.

Ministry of Health and Ministry of Environment. 2001. National Environmental Health Action Plan Lithuania. Vilnius: Ministry of Health.
Ministry of Health and Ministry of Environment Poland. 1999. National Environmental Health Action Plan for Poland. Warsaw: Ministry of Health.
Ministry of Housing, Spatial Planning and the Environment and Ministry of Health, Welfare and Sport. 2002. Action Plan Environmental Health: Implementing More Powerful Policy. The Hague: Ministry of Housing, Spatial Planning and the Environment.
State Committee of Environment of the Republic of Uzbekistan and Minister of Health of the Republic of Uzbekistan. 1999. National Environmental Health Action Plan for the Republic Of Uzbekistan. Tashkent: State Committee of Environment.
Swiss Federal Office of Public Health and Swiss Agency for the Environment Forests and Landscape. 1997. Sustainable Development: Action Plan Environment and Health. Bern: SFOPH.
Victorin K, Hogstedt C, Kyrklund T, Eriksson M. 1999. Setting priorities for environmental health risks in Sweden. In: Environmental Health for All: Risk Assessment and Risk Communication for National Environmental Health Action Plans (Briggs DJ, Stern R, Tinker TL, eds). Dordrecht, The Netherlands: Kluwer Academic Publishers, 35-52.
